# Down-regulation of vascular GLP-1 receptor expression in human subjects with obesity

**DOI:** 10.1038/s41598-018-28849-1

**Published:** 2018-07-13

**Authors:** Tomohiko Kimura, Atsushi Obata, Masashi Shimoda, Ikki Shimizu, Gabriela da Silva Xavier, Seizo Okauchi, Hidenori Hirukawa, Kenji Kohara, Tomoatsu Mune, Saeko Moriuchi, Arudo Hiraoka, Kentaro Tamura, Genta Chikazawa, Atsuhisa Ishida, Hidenori Yoshitaka, Guy A. Rutter, Kohei Kaku, Hideaki Kaneto

**Affiliations:** 1 Division of Diabetes, Endocrinology and Metabolism, Kawasaki Medical School, Kurashiki, Japan; 2 Department of Diabetes, Sakakibara Heart Institute, Okayama, Japan; 3 Section of Cell Biology and Functional Genomics, Department of Medicine, Imperial College London, London, UK; 4 Department of Cardiovascular Surgery, Sakakibara Heart Institute, Okayama, Japan; 5 Department of General Internal Medicine 1, Kawasaki Hospital, Kawasaki Medical School, Okayama, Japan

## Abstract

It has been thought that incretin signaling prevents arteriosclerosis, and very recently anti-arteriosclerotic effects through GLP-1 receptor were finally demonstrated in clinical human study. The purpose of this study was to investigate how vascular GLP-1 receptor expression is influenced in human subjects. First, we evaluated GLP-1 receptor expression in human arteries in immunostaining. Next, we separated the artery into the intima and media, and evaluated gene expression levels of various factors. We divided the subjects into obesity and non-obesity group and compared their expression levels between them. Finally, we evaluated which factors determine vascular GLP-1 receptor expression. GLP-1 receptor expression in intima and media was lower in obesity group compared to non-obesity group which was correlated with the alteration of TCF7L2 expression. Multiple regression analyses showed that BMI was an independent determining factor for GLP-1 receptor expression in the intima and media. Furthermore, using small interfering RNA method and TCF7L2-EGFP adenovirus, we showed that TCF7L2 was involved in GLP-1 receptor expression in human vascular cells. Taken together, vascular GLP-1 receptor and TCF7L2 expression was significantly down-regulated in human subjects with obesity. In addition, it is likely that TCF7L2 functions as a modulator of vascular GLP-1 receptor expression.

## Introduction

It is known that GLP-1 receptor is present not only in the pancreas but also in various tissues including the heart, blood vessels, central nerve, vagus nerve, stomach, intestines, visceral fat and the kidney^1,2^. GLP-1 signaling in pancreatic β-cells increases insulin secretion in a blood glucose level-dependent manner, leading to decrease of blood glucose levels^3^. At the hypothalamus, its stimulation induces loss of appetite, leading to weight loss^3,4^. It is also known that incretin signal improves vascular relaxation response through eNOS expression and activity in endothelial cells^5^. GLP-1 signaling suppresses the expression of various pro-atherosclerotic factors in vascular endothelial cells induced by hyperglycemia and inflammatory cytokines (e.g. TNF-α)^6,7^. Moreover, it is known that in arteriosclerosis model mice, GLP-1 signaling increases the expression of eNOS which leads to the dilatation of blood vessel^8^. In addition, it was reported that intravenous GLP-1 receptor agonist administration improved flow-mediated dilatation in human subjects^9^. Considering from these reports, vascular GLP-1 signaling improves blood vessel wall abnormalities induced by various factors such as hyperglycemia and various inflammatory cytokines^10–12^. In addition, it is known that GLP-1 signaling has anti-atherosclerotic effects in vascular smooth muscle cells^13–16^. Anti-arteriosclerotic effects through GLP-1 receptor have been indicated so far only in basic experiments such as animal experimental model and/or cell culture system. But finally, this effect was also demonstrated very recently in the clinical human study. It was shown that in patients with type 2 diabetes, the usage of GLP-1 receptor agonist resulted in reduction of cardiovascular-related events and/or death^17^. Accordingly, the role of GLP-1 receptor in blood vessels has attracted much attention recently.

On the other hand, it has been reported that GLP-1 receptor expression in β-cells is decreased in the diabetic state^18,19^ and that TCF7L2 functions as a transcription factor of GLP-1 receptor at least in β-cells^20^. However, it remains unclear whether vascular GLP-1 receptor expression changes under some conditions and which factors could affect vascular GLP-1 receptor expression. The purpose of this study was to reveal the alteration of vascular GLP-1 receptor expression and the factors affecting its expression in human subjects.

## Methods

### Study population

The study population consisted of 40 patients who needed artery surgery and were admitted to the department of cardiovascular surgery of the Sakakibara Heart Institute from October 2014 to April 2015. The study protocol was reviewed and accepted by the both hospital ethics committee of Sakakibara Heart Institute and Kawasaki Medical School (No. 1933). And the study protocol registered to UMIN-CTR (No.UMIN000024556) approved by International Committee of Medical Journal Editors (ICMJE). We recruited the subjects who were ≥20 years old and agreed to participate in this study. Written informed consent to participate in this study was obtained from all subjects or their representatives. We excluded the subjects having some endocrine disease and/or severe mental disorder. Basically, we performed the recruiting of subjects who met the inclusion criteria within a study period. All methods were performed in accordance with the relevant guidelines and regulations.

### Blood sampling and section preparation

Blood was collected as much as possible when the condition was sufficiently stable after operation. Resected disease artery was immersed in RANlater (Qiagen, Venlo, Netherlands) and stored until use. Excised diseased artery was mechanically separated by forceps into the intima and media as described previously^21^.

### Immunostaining

The sections were embedded in paraffin. According to a previously reported method^22^, immunostaining was performed using anti-hGLP-1 receptor antibody (MAb 3F52, Novonordisk Pharma Ltd, Copenhagen, Denmark). The sections were heated for 15 min in a microwave for antigen retrieval, and endogenous peroxidase activity was blocked by immersion in 1% (vol./vol.) H2O2 for 15 min. After washing in TBST, sections were blocked with 1% skimmed milk for 15 min, and then incubated with anti-hGLP-1 receptor antibody at room temperature for 14 h. After rinsing with TBST, an adequate amount of anti-mouse immunoglobulin-HRP (DAKO) was added, and the reaction was allowed to proceed at 25 °C for 30 min. After another washing in TBST, an adequate amount of amplification reagent (DAKO) was added, and the reaction was allowed to proceed at 25 °C for 5 min. In order to do an immunostaining the vascular intima, isolectin GS-IB4 Alexa Fluor 594 conjugate (Invitrogen) was applied to the sections. After rinsing with PBST, DAPI (SIGMA-ALDRICH) was put for 5 min at room temperature. We performed TCF7L2 staining according to a previously reported method^23^. Antibody for anti-TCF7L2 antibody (abcam) and anti-CD34 antibody (Santa Cruz Biotechnology) was using as primary antibody. Then, we added a mixture of the second antibodies (Alexa Fluor 488 donkey anti-Goat IgG and Alexa Fluor 594 donkey anti-mouse IgG; Invitrogen).

### Real-time PCR

We mechanically separated excised diseased artery into the intima and media, and evaluated mRNA levels of various factors in the intima and media by real time RT-PCR. We prepared primer pairs encoding genes related to GLP-1 receptor and arteriosclerosis-related factors, and performed real-time RT-PCR using Sybr Green. To quantify gene expression, the 2^−ΔCT^ was calculated using β-actin as an internal control.

### Human umbilical vein endothelial cells (HUVEC) and human aortic endothelial cells (HAEC) and small interfering RNA (siRNA)

HUVEC and HAEC were purchased from Lonza (USA). The cells were cultured in Endothelial Cell Growth Medium 2 (EGM2 BulletKit, CC3162; Lonza) in 5% CO2 at 37 °C, separately. Before the experiment, the cells were incubated for 24 hours or until enough amount could be secured for experiment. We exposed HUVEC and HAEC to siRNA directed to TCF7L2 (siTCF7L2) or scrambled siRNA as a control and cultured them for 24 hours. After then, real-time RT-PCR using Sybr Green and western blotting were performed.

### Overexpression of TCF7L2 in HAEC using TCF7L2-EGFP adenovirus

TCF7L2-EGFP adenovirus was prepared as described previously^24^. HAEC were incubated in EGM2 until enough amount could be secured for experiment. We exposed HAEC to TCF7L2-EGFP adenovirus or control EGFP and cultured them for 48 hours. After then, real-time RT-PCR with Sybr Green and western blotting were performed.

### Statistical analysis

Subjects were divided into obesity (BMI ≥ 25 kg/m^2^) and non-obesity group (BMI < 25 kg/m^2^) and comparison of the clinical parameters and various gene expression levels in the intima and media was performed. The results were expressed as mean ± SE. A Wilcoxon test was used to test the difference between obesity and non-obesity group with p < 0.05 regarded as significant. χ^2^ test was used for the comparison of obese- and non-obese subjects at the entry point and the comparison of various parameters between the presence and absence of various factors. To examine which factors are associated with GLP-1 receptor expression in human aorta, spearman’s rank correlation coefficient test was performed. Furthermore, to examine which factors independently determine GLP-1 receptor expression in the intima and media, we performed multiple regression analysis.

## Results

### Characteristics of the study subjects

The background characteristics of 40 subjects in this study were as follows: Male/female =27/13, abdominal or thoracic aortic aneurysm/aortic dissection/arteriosclerosis obliterans/other = 12/20/6/2, diabetic/non-diabetic = 13/26, age 68.0 ± 13.4 years old, BMI 23.8 ± 4.7 kg/m^2^, HbA1c 6.2 ± 1.5%. BMI in obese (BMI ≥ 25 kg/m^2^, n = 16) and non-obese group (BMI < 25 kg/m^2^, n = 24) was 28.6 ± 2.5 kg/m^2^ and 20.7 ± 2.8 kg/m^2^, respectively (p < 0.05) (Table 1). There was no significant difference in fasting plasma glucose (121.2 ± 32.2 vs 116.6 ± 25.0 mg/dl), HbA1c (5.9 ± 0.6 vs 6.4 ± 1.9%), HDL-cholesterol (37.1 ± 10.3 vs 39.0 ± 9.0 mg/dl), LDL-cholesterol (111.1 ± 38.1 vs 91.3 ± 33.0 mg/dl) between the obesity and non-obesity group (Table 1).

**Table 1:** Patient background-Obese and non-obese subjects-.

	Obese (n = 16) (BMI ≥ 25 kg/m^2^)	Non-obese (n = 24) (BMI < 25 kg/m^2^)	*p*
Arterial dissection/Aneurysm/ ASO/Amputation/etc.	11/5/0/0/0	9/7/6/1/1	
Diabetes/Non-diabetes	5/11 (31.3%)	6/17 (26.1%)	n.s.
With hypertension	5 (31.3%)	7 (29.2%)	n.s.
With ARB or ACE-I	4 (25.0%)	3 (12.5%)	n.s.
With Ca-blockers	3 (18.8%)	4 (16.7%)	n.s.
With dyslipidemia	13 (81.3%)	19 (79.2%)	n.s.
With statin	3 (18.8%)	8 (33.3%)	n.s.
With anti-coagulant drugs	0 (0%)	4 (16.7%)	0.036
Age	61.4 ± 16.7	72.1 ± 9.1	0.047
BMI (kg/m^2^)	28.6 ± 2.5	20.7 ± 2.8	<0.0001
HbA1c (%)	5.9 ± 0.6	6.4 ± 1.9	n.s.
Fasting plasma glucose (mg/dl)	121.2 ± 32.2	116.6 ± 25.0	n.s.
LDL cholesterol (mg/dl)	111.1 ± 38.1	91.3 ± 33.0	n.s.
HDL cholesterol (mg/dl)	37.1 ± 10.3	39.0 ± 9.0	n.s.
C-reactive protein (mg/dl)	2.3 ± 1.0	1.9 ± 0.6	n.s.

Abbreviations: ARB, angiotensin II receptor blocker; ACE-I, angiotensin converting enzyme inhibitor; ASO, arteriosclerosis obliterans; BMI, body mass index; LDL, low-density lipoprotein; HDL, high-density lipoprotein; n.s. not significant.

### Evaluation of GLP-1 receptor and TCF7L2 expression in the aorta of human subjects in immunostaining: Down-regulation of GLP-1 receptor and TCF7L2 expression in obese subjects

**Figure 1:**
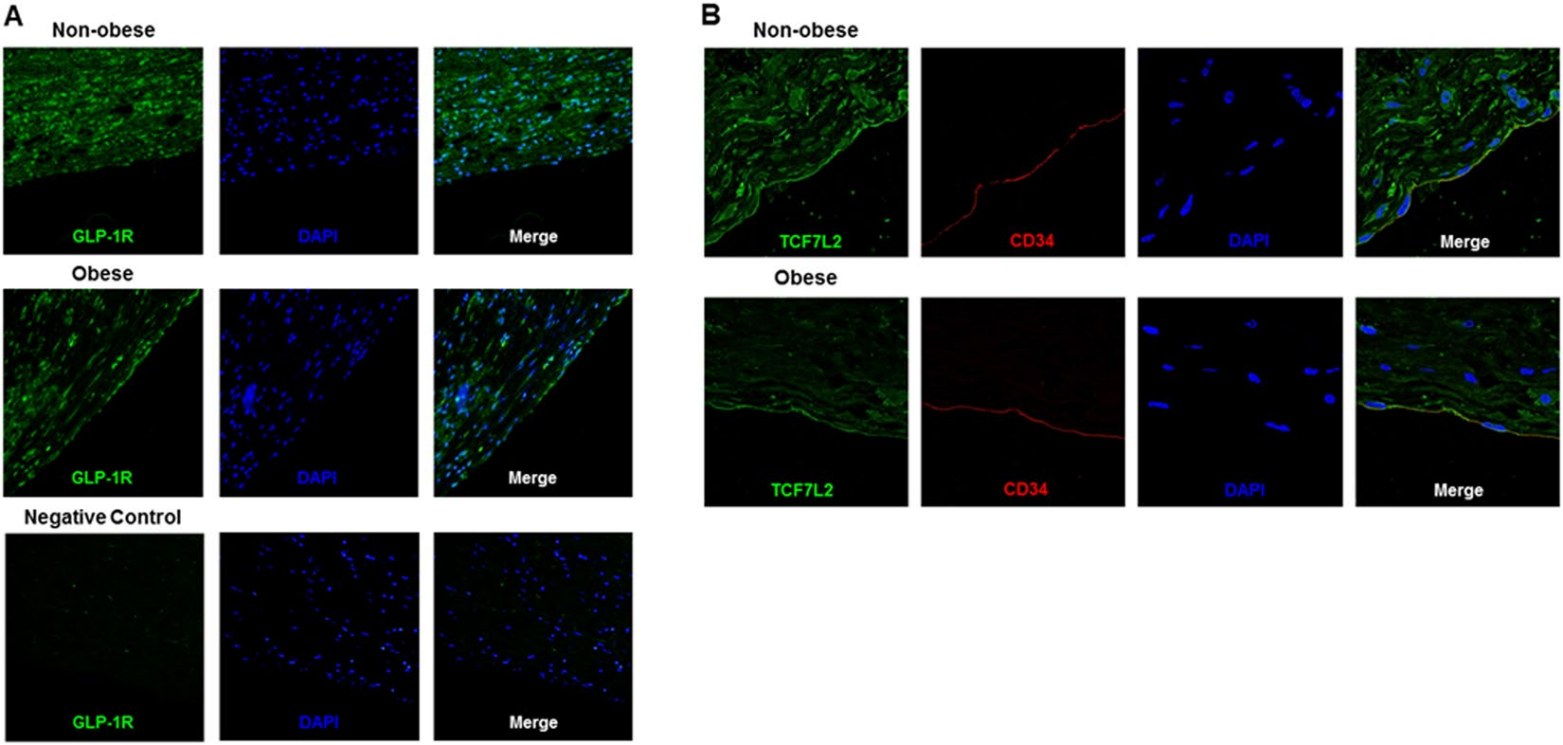
(**A**) Comparison of vascular GLP-1 receptor expression between non-obese (upper panels) and obese human subjects (middle panels). Double staining for GLP-1 receptor in green, DAPI in blue in human artery sections. Lower panels show immunostaining for GLP-1 receptor without the GLP-1 receptor antibody. (**B**) Comparison of vascular TCF7L2 expression between non-obese and obese human subjects. Triple staining for TCF7L2 in green, intima in red with CD34 antibody, DAPI in blue in human artery sections.

Although most of the GLP-1 receptor antibodies available now are not working very well in several experiments, it has been drawing attention that GLP-1 receptor antibody MAb 3F52 which was developed by Novonordisk Pharma has been working very well^22^. Therefore, in the present study, we evaluated GLP-1 expression in human aorta using this antibody. First, to examine GLP-1 receptor expression in human aorta, immunostaining was performed with this excellent antibody specific for GLP-1 receptor. As shown in Fig. 1A, GLP-1 receptor expression in the vascular intima of human was confirmed as observed in other animals^22^. Furthermore, the expression of GLP-1 receptor in obese subjects (middle panels) was much lower compared to non-obese subjects (upper panels). In order to examine the quality of GLP-1 receptor antibody and to show that such staining is not non-specific, we performed immunostaining in the same way without this antibody. As shown in lower panels, without adding this GLP-1 receptor antibody, there was no staining at all. Similarly, to examine TCF7L2 expression in human aorta, immunostaining was performed with antibody for TCF7L2. As shown in Fig. 1B, the expression of TCF7L2^23^ in the intima and media was clearly lower in obese subjects (lower panel) compared to non-obese subjects (upper panel).

### Down-regulation of GLP-1 receptor and TCF7L2 mRNA expression both in the intima and media of arteries in obese human subjects

**Figure 2:**
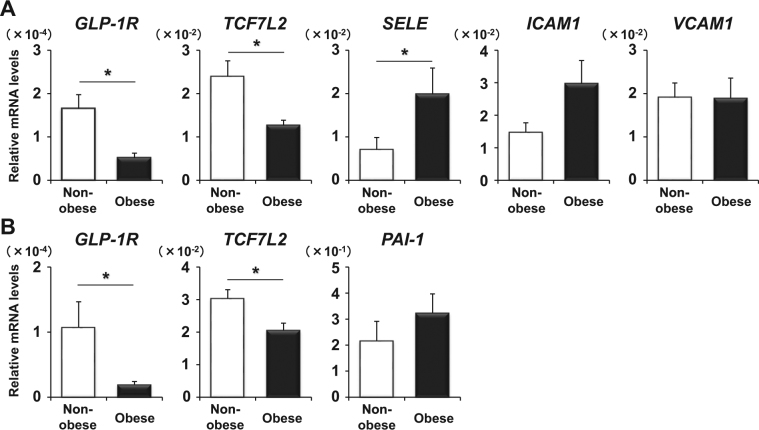
(**A**,**B**) Expression levels of various genes associated with arteriosclerosis in the intima (GLP-1 receptor, TCF7L2, selectin, ICAM1, VCAM1) (**A**) and arteriosclerosis-related genes in the media (GLP-1 receptor, TCF7L2, PAI-1) (**B**) in non-obese (white) and obese human subjects (black). Data are presented as mean ± S.E. n (Obese/Non-obese = 16/24), *p < 0.05.

Since we obtained the findings suggesting that GLP-1 receptor protein expression in obese subjects is lower compared to that in non-obese subjects, we mechanically separated the excised diseased artery into the intima and media and quantitatively evaluated various gene expression levels in the intima in the obesity and non-obesity group. As shown in Fig. 2A, GLP-1 receptor and TCF7L2 mRNA levels in the intima were significantly lower in obesity group compared with that in non-obesity group (p < 0.01 and p = 0.03, respectively), which was consistent with the immunostaining results. In contrast, mRNA level of E-selectin which is involved in the adhesion of leukocytes and vascular endothelial cells was significantly higher in obesity group compared to non-obesity group (*p* = 0.02). ICAM1 mRNA level tended to be higher in obesity group compared to non-obesity group, but it did not reach a significant difference. Similarly, we compared expression levels of various factors between obesity and non-obesity group in the media. As shown in Fig. 2B, GLP-1 receptor and TCF7L2 mRNA levels in the media were significantly lower in obesity group compared to non-obesity group (p < 0.01 and p = 0.02, respectively) which was also consistent with the immunostaining data. In contrast, PAI-1 mRNA level in obesity group tended to be higher than that in non-obesity group. It is noted here that although it is known that TCF7L2 functions as a transcriptional factor of GLP-1 receptor gene in pancreatic β-cells^20^, it is not thought that ICAM1 and PAI-1 are the downstream of TCF7L2.

### TCF7L2 is a possible regulator of GLP-1 receptor expression in human vascular cells

**Figure 3:**
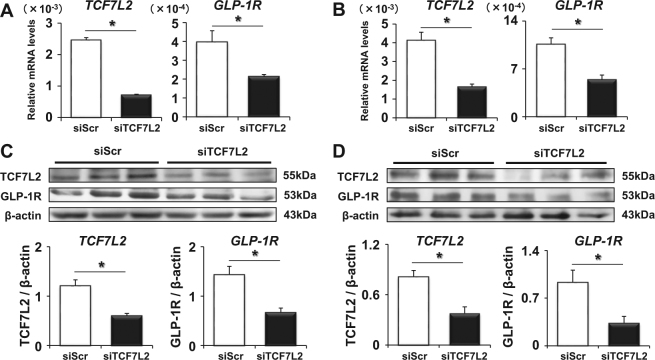
TCF7L2 and GLP-1 receptor mRNA (**A**) and protein expression levels (**C**) in HUVEC after the cultivation with scrambled control (siScr) or TCF7L2 siRNA (siTCF7L2). TCF7L2 and GLP-1 receptor mRNA (**B**) and protein expression levels (**D**) in HAEC after the cultivation with siScr or siTCF7L2. Data are shown as mean ± S.E. n = 10, *p < 0.05.

**Figure 4:**
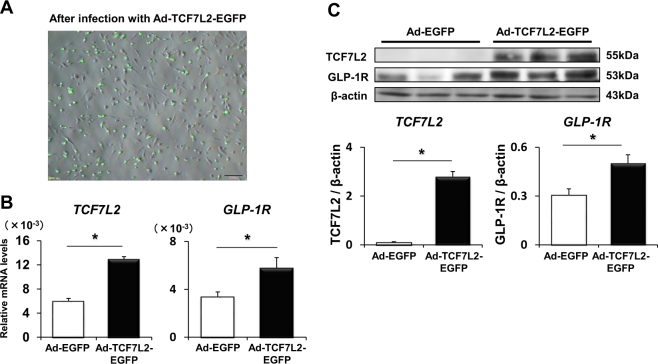
(**A**) Overexpression and subcellular localization of TCF7L2-EGFP in HAEC was assessed by microscopy 48 hours after infection of TCF7L2-EGFP expressing adenovirus (Ad-TCF7L2-EGFP). (**B**,**C**) TCF7L2 and GLP-1 receptor mRNA (**B**) and protein levels in HAEC (**C**) after the treatment with control adenovirus (Ad-EGFP) or Ad-TCF7L2-EGFP. Data are presented as mean ± S.E. n = 8 (**A**,**B**), n = 5 (**C**) *p < 0.05.

Since there was correlation between the expression levels of GLP-1 receptor and TCF7L2 in the aorta in our experiments and it is well known that TCF7L2 is a transcriptional factor of GLP-1 receptor in pancreatic β-cells, we hypothesized that TCF7L2 regulates GLP-1 receptor expression in human vascular cells as observed in β-cells. To demonstrate this hypothesis, we exposed human umbilical vein endothelial cells (HUVEC) and human aortic endothelial cells (HAEC) to siRNA directed to TCF7L2 (siTCF7L2) or scrambled control siRNA and cultured them for 24 hours. Both in HUVEC (Fig. 3A) and HAEC (Fig. 3B), siTCF7L2 significantly reduced TCF7L2 mRNA levels compared to control, and such reduction of TCF7L2 led to the significant down-regulation of GLP-1 receptor mRNA expression level, respectively (p = 0.0005, p = 0.0024). Since it is important to know that protein levels of TCF7L2 and GLP-1 receptor are also altered after the change of their mRNA levels, we performed western blotting to confirm this point. As shown in (Fig. 3C,D), both in HUVEC (Fig. 3C) and HAEC (Fig. 3D), siTCF7L2 decreased TCF7L2 protein levels compared with control and such reduction of TCF7L2 led to the significant down-regulation of GLP-1 receptor protein expressions, respectively (*p* = 0.005, *p* = 0.002). Furthermore, in order to strengthen our hypothesis, we conducted the TCF7L2 overexpression study using TCF7L2-EGFP expressing adenovirus^24^. We exposed HAEC to TCF7L2-EGFP adenovirus or control EGFP and cultured them for 48 hours. As expected, TCF7L2-EGFP expression was largely confined to the nucleoplasm (Fig. 4A). As shown in Fig. 4B, TCF7L2-EGFP adenovirus significantly increased TCF7L2 mRNA levels compared with control and such increment of TCF7L2 led to the significant up-regulation of GLP-1 receptor expressions (*p* < 0.05). A similar phenomenon was proved even with protein; TCF7L2-EGFP adenovirus significantly increased TCF7L2 and GLP-1 receptor protein expression levels as well (Fig. 4C, *p* = 0.04).

### Body mass index (BMI) is an independent determining factor of GLP-1 receptor expression both in the intima and media of aorta in human subjects

Next, to examine which factors are associated with GLP-1 receptor expression in human aorta, we performed univariate analyses. As shown in Table 2, there was significantly negative correlation between GLP-1 receptor gene expression and BMI, LDL-cholesterol in the intima (*p* < 0.01 and p = 0.02, respectively) and media (*p* < 0.001 and p = 0.04, respectively). Furthermore, to examine which factors independently determine GLP-1 receptor expression in the intima and media, we performed multivariate analyses. As shown in Table 2, only BMI had a significant negative correlation with GLP-1 receptor gene expression in the intima and media (p = 0.03 and p < 0.001, respectively).

**Table 2:** The independent factor which contributes to GLP-1 receptor expression.

Clinical parameter	Univariate analysis		Multivariate analysis	
	ρ	*p*	β	*p*
Vascular intima				
Age	0.14	n.s.	−0.14	n.s.
Gender	−0.06	n.s.	0.08	n.s.
BMI	−0.41	<0.01	−0.40	<0.05
HbA1c	−0.14	n.s.		
Fasting plasma glucose	0.02	n.s.		
LDL cholesterol	−0.37	<0.05	−0.24	n.s.
HDL cholesterol	−0.10	n.s.		
Vascular media				
Age	0.20	n.s.	−0.02	n.s.
Gender	−0.16	n.s.	0.13	n.s.
BMI	−0.55	<0.001	−0.56	<0.001
HbA1c	0.00	n.s.		
Fasting plasma glucose	0.32	n.s.		
LDL cholesterol	−0.32	<0.05	−0.14	n.s.
HDL cholesterol	0.02	n.s.		

Abbreviations: BMI, body mass index; LDL, low-density lipoprotein; HDL, high-density lipoprotein; n.s. not significant.

We examined the possible correlation of GLP-1 receptor and TCF7L2 mRNA levels with the type of arterial pathology observed. However, there was no clear correlation between them. In addition, we compared GLP-1 expression between subjects with and without various diseases or medication. However, between the subjects with and without hypertension (*p* = 0.16 and *p* = 0.52, respectively), dyslipidemia (*p* = 0.51 and *p* = 0.42, respectively) or diabetes (*p* = 0.88 and *p* = 0.68, respectively), there were no significant differences in GLP-1 receptor expression in intima and media, respectively. There were no significant difference in GLP-1 receptor expression in intima and media between the subjects with and without ARB (*p* = 0.83 and *p* = 0.75, respectively) or anti-diabetic drugs (*p* = 0.70 and p = 0.20, respectively). Also, there was no difference in GLP-1 receptor expression between young and senior subjects (*p* = 0.14 and p = 0.17, respectively) (≥65 years old) and between subjects with and without urinary albumin (*p* = 0.55 and *p* = 0.37, respectively) (≥30 mg/gCr).

## Discussion

It has been reported so far that incretin plays pleiotropic roles in various kinds of tissues and many scientists all over the world have been performing a variety of experiments to elucidate the importance of incretin signaling. In such a situation, one of the largest obstacles is that most of the GLP-1 receptor antibodies which are available now are not working very well in several experiments including immunostaining and western blotting. It has been drawing attention, however, GLP-1 receptor antibody MAb 3F52 which was developed by Novonordisk Pharma has been working very well^22^. Indeed, various studies using this excellent antibody has showed the localization of GLP-1 receptor and novel findings in primates one after another. It is no exaggeration to say that the appearance of the antibody has repainted the conventional idea. This excellent antibody was working very well in this study as reported^22^ and thereby enabled us to start performing this research and to demonstrate the new findings in human subjects.

In the present study, we demonstrated GLP-1 receptor expression in human artery was down-regulated in subjects with obesity and/or hypercholesterolemia. Generally, obese subjects tend to have higher frequency to cause macroangiopathy compared to non-obese subjects. Therefore, we think that declining of GLP-1 receptor expression in aorta in obese subjects may be involved in such high frequency of macrovascular complications. It has been thought that vascular GLP-1 signaling acts as anti-arteriosclerotic effects. But there was no report showing the factors that contribute to GLP-1 receptor expression in human artery. To the best of our knowledge, this is the first report demonstrating that GLP-1 receptor expression decreases in the aorta of obese human subjects. It was reported that GLP-1 receptor expression in pancreatic β-cells was suppressed by gluco-lopotoxicity^18,19^. Considered from the data in this study, lipotoxicity rather than hyperglycemia is likely related to the reduced expression of GLP-1 receptor in aorta, although it is necessary to perform further study in order to strengthen our hypothesis. It seems that there is some difference between acute and chronic effects of increased glucose and/or cholesterol levels on GLP-1 receptor expression levels. Since it was practically difficult to culture HUVEC for a long period, we cultured HUVEC for 3–5 days under increased glucose and/or cholesterol levels and then evaluated GLP-1 receptor expression levels. As the results, interestingly, GLP-1 receptor expression was not decreased at all after acute exposure to increased glucose and/or cholesterol levels (Supplemental Fig. 1). However, as shown in this study, GLP-1 receptor expression was significantly decreased in obese subjects. Therefore, we assume that there is some large difference between acute and chronic effects of increased glucose and/or cholesterol levels on GLP-1 receptor expression levels. Indeed, it is well known that there is large difference between acute and chronic effects of various stimuli on some phenomena. For example, acute exposure to high glucose concentration increases GIP receptor expression in pancreatic β-cells whereas chronic exposure to high glucose concentration reduces GIP receptor in β-cells^19^. Although speculative, we assume that after short exposure of artery to hyperglycemia and/or hypercholesterolemia, GLP-1 receptor increases to protect artery initially, but that after chronic exposure to them, such protective pathway does not appropriately function. In addition, it was strongly suggested that TCF7L2 is the modulator of GLP-1 receptor in human endothelial cells as observed in β-cells^20^. It was reported that TCF7L2 plays a cell autonomous role in the control of pancreatic β-cell function and mass^25^. We made TCF7L2 expressing adenovirus^24^ and confirmed that TCF7L2 overexpression increased GLP-1 receptor expression in arterial cells. These data showed the possible coordination between TCF7L2 and GLP-1 receptor in arterial cells. In addition, since TCF7L2 is a transcription factor, we think that TCF7L2 trans-activates GLP-1 receptor gene in cell autonomous manner. It has been reported that GLP-1 plays protective role on the progression of atherosclerosis. Therefore, we think the preservation of TCF7L2 level in the arterial cells would be very important for the prevention and/or protection of atherosclerosis.

As shown in Table 2, both BMI and LDL cholesterol were associated with GLP-1 receptor expression in univariate analysis, but in multivariate analysis BMI alone was significant determinant factor for GLP-1 receptor expression level and LDL cholesterol became non-significant for it. Since there could be some co-linearrity between BMI and LDL cholesterol in several cases, we think that the non-significance of LDL cholesterol in multivariate analysis might have been due to such possible co-linearity between them.

In addition, it was previously reported that GLP-1 signaling activates endothelial nitric oxide synthase (eNOS) and reduces reactive oxygen species (ROS) which finally leads to reduce inflammatory process and the progression of atherosclerosis. Therefore, we assume that the decreased expression of arterial GLP-1 receptor in obese subjects would lead to the inactivation of eNOS and increase of ROS. We also think that such decreased GLP-1 receptor expression could explain, at least partially, the mechanism by which atherosclerosis is facilitated in obsess subjects. In addition, at present there was no report demonstrating that TCF7L2 *per se* had an anti-inflammatory effect. However, if it is demonstrated that TCF7L2 has some anti-inflammatory effects, GLP-1 receptor could be a crucial down-stream event for TCF7L2-mediated anti-inflammatory effects. Therefore, it is possible that preservation of TCF7L2 expression could lead to reduce the atherosclerosis through the increase of GLP-1 receptor expression and decrease of inflammation process, although further evaluation would be necessary to demonstrate this point.

There is a limitation in this study. Because it is very difficult to obtain arteries in subjects who do not need surgical operation, we performed various experiments using large and small arteries in subjects with some vascular diseases which need surgical operation. Therefore, we cannot exclude the possibility that the data in this study were partially influenced by the presence of such vascular diseases. In addition, it would be better to perform western blotting rather than immunohistochemistry in order to perform quantitatively evaluation. Although we performed western blotting and the convincing data in HUVEC and HAEC experiments, we failed to obtain the patient samples for western blotting because many patients in this study needed emergency operation. Next, it would be much better to show some metabolic parameters involved in regulating arterial GLP-1 receptor and TCF7L2 levels. Since it has been reported that GLP-1 receptor expression level in pancreatic β-cells is down-regulated by lipotoxicity such as free fatty acids (FFA) and/or triglyceride (TG) both of which are usually high in obese subjects, we assume that FFA and TG are possible parameters involved in arterial GLP-1 receptor and TCL7L2 levels. On the other hands, it is well known that there is marked difference between before and after meal in such parameter levels. Since many patients in this study needed emergency operation, we failed to obtain such parameters in fasting status. In addition, since many patients needed intravenous drip injection after the emergency operation, FFA and TG levels were markedly affected by such injection. In the present study, we showed that GLP-1 receptor was present in the intima and media and that its expression level was lower in obese subjects. However, it is known that GLP-1 receptor is present in various tissues in vessels such as macrophages and monocytes. In addition, it has been drawing attention that GLP-1 acts on GLP-1 receptor in atrium which contributes to the relaxation of blood vessels. Therefore, it remains unclear which GLP-1 receptor expression is most important for such relaxation of blood vessels among GLP-1 receptor expression in various kinds of cells.

Taken together, we think that the findings in the present study using vascular cells in human subjects would provide marvelous information and have great significance not only from the clinical point of view but also in vascular biology research area, because very recently anti-arteriosclerotic effects through GLP-1 receptor were demonstrated in clinical human study in addition to basic study. Since it was shown that administration of GLP-1 receptor agonist in subjects with type 2 diabetes resulted in reduction of cardiovascular-related events and/or death^17^, the role of GLP-1 receptor in vascular cells has been drawing much more attention in clinicians as well as researchers. In such circumstances, we believe that the decrement of vascular GLP-1 receptor expression is involved in the progression of arteriosclerosis and the onset of cardiovascular events. In addition, the data in this study suggest the possibility that incretin-related drugs such as GLP-1 receptor agonist would exert more protective effects on arteries in non-obese subjects and that amelioration of obesity would be beneficial to augment the protective effects of such drugs on arteries although further evaluation such as prospective clinical study would be necessary to demonstrate these points.

## Electronic supplementary material

Supplementary Material
