# Supplementary material for: Down-regulation of vascular GLP-1 receptor expression in human subjects with obesity
Source: Sci Rep. 2018 Jul 13;8:10644. doi: 10.1038/s41598-018-28849-1 (PMC6045606; doi:10.1038/s41598-018-28849-1)

Down-regulation of vascular GLP-1 receptor expression in human subjects with obesity

Tomohiko Kimura, Atsushi Obata, Masashi Shimoda, Ikki Shimizu, Gabriela da Silva Xavier, Seizo Okauchi, Hidenori Hirukawa, Kenji Kohara, Tomoatsu Mune, Saeko Moriuchi, Arudo Hiraoka, Kentaro Tamura, Genta Chikazawa, Atsuhisa Ishida, Hidenori Yoshitaka, Guy A. Rutter, Kohei Kaku, Hideaki Kaneto

SUPPLEMENTARY FIGURE

Supplementary Figure 1. HUVEC were cultured with various concentrations of glucose and/or palmitate for 72 hours. Upper and lower panels show TCF7L2 and GLP-1 receptor mRNA expression levels, respectively, 72 hours after the treatment. Data are presented as mean±S.E. n = 5

Supplemental Figure 1

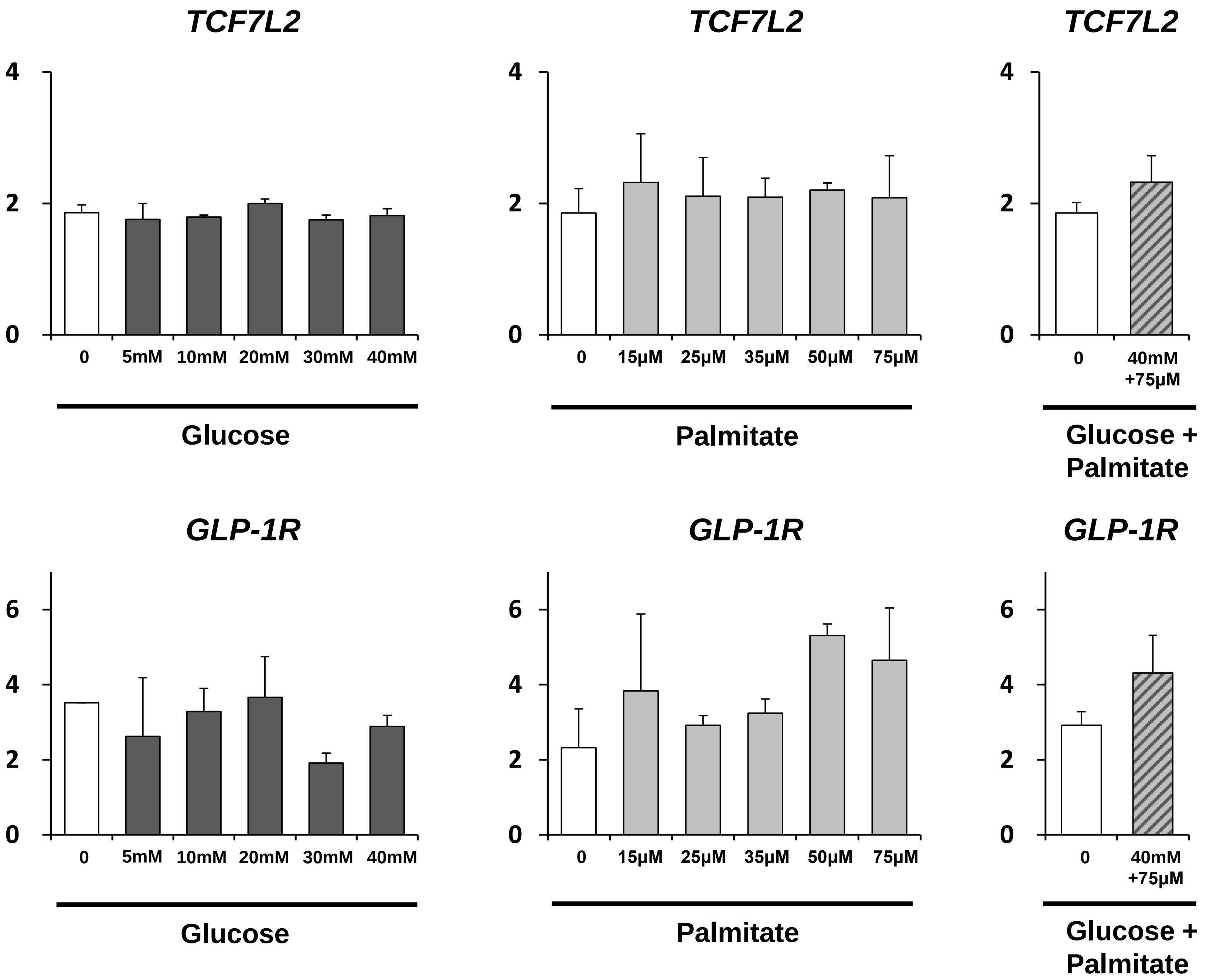

Supplement: Supplementary file 1 — Supplementary Material [file 41598_2018_28849_MOESM1_ESM.pdf]
